# The role of brokers in cultivating an inter-institutional community around open educational resources in higher education

**DOI:** 10.1007/s10734-022-00876-y

**Published:** 2022-06-03

**Authors:** Marjon Baas, Robert Schuwer, Ellen van den Berg, Tjark Huizinga, Roeland van der Rijst, Wilfried Admiraal

**Affiliations:** 1 ICLON-Graduate School of Teaching, Leiden University, Kolffpad 1, Leiden, 2333BN The Netherlands; 2 School of Education, Saxion University of Applied Sciences, Van Galenstraat 20, Enschede, 7511JL The Netherlands; 3 School of ICT, Fontys University of Applied Sciences, Rachelsmolen 1, Eindhoven, 5612MA The Netherlands; 4 Centre for the Study of Professions, Oslo Metropolitan University, Pilestredet 40, N-0170 Oslo, Norway

**Keywords:** Brokers, Boundary spanning, OER, CHAT, Communities, Higher education

## Abstract

Brokers are individuals who facilitate transfer of knowledge and resources, and coordinate efforts across boundaries of organizations. They are defined by their role rather than their organizational position. Brokers might be imperative for the formation and maintenance of inter-institutional relationship as they have the responsibility and the necessary structural position to connect otherwise separate groups. In the context of this study, brokers had the role to cultivate an inter-institutional community around open educational resources (OER) by connecting groups of teachers across higher education institutes. OER provide higher education institutes with an aid to face the challenges of improving teaching and learning. Yet most OER users encounter challenges that relate to finding resources that are relevant, up-to-date, and of good quality. Communities could minimize this issue, but many OER initiatives fizzle out as expanding their impact is an arduous task. This qualitative descriptive study draws upon cultural-historical activity theory to understand the complexities associated with the role of brokers in creating sustainable collaboration on OER across 15 higher education institutes in the Netherlands. Data was collected from project documents, process reports, reflections reports, and a retrospective focus group. The findings show that brokers engaged in a wide variety of actions but that a small-scale, personal, and content-oriented approach to encourage teachers to engage with the OER repository and the online community was perceived as the most valuable. Brokers also experienced conflicts due to the demanding context they were operating in, the ambiguity of their role, and the organizational constraints they were confronted with. Practical implications refer to supporting higher education institutes that wish to initiate sustainable collaboration across institutes.

**Supplementary Information:**

The online version contains supplementary material available at 10.1007/s10734-022-00876-y.

## Introduction

The number of open educational resources (OER) available in online repositories is ever-growing. Due to their unique characteristics, teachers may retain, reuse, revise, remix, and redistribute these resources (Wiley, 2014) allowing them to adapt OER to their teaching needs (Belikov & Bodily, 2016). OER could support initiatives to improve teaching and learning (Orr et al., 2015), for example by improving access to student learning by reducing costs (Hilton et al., 2014), improving teachers’ critical reflection on their practices (Weller et al., 2015), or increasing collaboration between teachers across institutes (Chae & Jenkins, 2015). Despite the potential of OER and the vast number of these resources available, adoption remains limited (Schuwer & Janssen, 2018). Several barriers have impeded adoption (Cox & Trotter, 2017), but a major problem for most OER users relates to finding resources that are relevant, up-to-date, and of good quality (Admiraal, 2022). Some researchers suggest that communities could minimize this issue (Baas et al., 2019; Clements & Pawlowski, 2012; Orr et al., 2015). Nonetheless, keeping activities using OER sustainable over a long period of time is essential for impacting teaching practice, yet most OER initiatives cease to exist after the initial project funding due to challenges relating to a central control of OER quality and increasing the size of the user group (Orr et al., 2015). Growing a small community of volunteers into a broader audience is arduous as it requires continuous collaboration across institutes to increase the size of the user group, despite the sociocultural differences that may exist between them (Akkerman & Bakker, 2011). Coordinators play an important role in this critical aspect of cultivating the user group, especially within distributed communities in which ties need to be established to connect several local groups into one community (Wenger et al., 2002). Brokers is a term often used to denote these coordinators who act as a bridge between sites such as across higher education institutes (Akkerman & Bakker, 2011). For example, in inter-institutional collaboration, sociocultural differences between institutes need to be overcome to avoid discontinuity of interaction in the longer term. It are brokers, who are individuals working within the institutes, that take up the role to facilitate boundary crossing by introducing elements of one practice into another. Brokers have a valuable yet difficult role with regard to spanning boundaries, yet limited knowledge is available to understand the particular complexities associated with the role of brokers in creating sustainable collaboration across institutes in higher education. Thus, the aim of this descriptive qualitative study was to contribute insights into the role of brokers in cultivating an inter-institutional community around OER.

## Theoretical framework

### Boundary spanning and the role of the broker

Although some great examples of sustainable OER communities do exist (e.g. MERLOT, OER Commons), studies on cultivating such communities are scarce. Even though a number of studies have described the design and outcomes of inter-institutional communities around OER (Borthwick & Dickens, 2013; Burgos-Aguilar & Mortera-Gutierrez, 2013; Tosato & Bodi, 2011), they do not provide any information about the persons spanning the boundaries between institutes to cultivate the community. Boundary spanners are essential, however, to the formation and maintenance of inter-institutional relationships through which the interdependency is managed (Van Meerkerk & Edelenbos, 2018). Due to their key role, we are specifically interested in these boundary spanners who have the responsibility and the necessary structural position to connect otherwise separate groups (Akkerman & Bruining, 2016). When connecting these separate groups, boundary spanners will encounter boundaries which ‘typically become visible and articulated when actors try to access something on the other side of the boundary and encounter obstacles or constraints in this quest’ (Engeström & Sannino, 2021, p. 21). How do boundary spanners span these boundaries? They apply a range of activities (Van Meerkerk & Edelenbos, 2018; Williams, 2002) as they (1) develop and maintain relationships on both a formal and informal and personal level to connect otherwise separate groups; (2) align, coordinate, and maintain activities and processes within both their own organization and the wider network; (3) mediate the information flow between organizations by both transferring information across boundaries and transforming information so that it can be understood across organizations; and (4) proactively respond to opportunities to exploit the collaboration and solve problems or to bend problems to solutions. What makes a boundary spanner successful? Besides these individual determinants that are often reported to impact boundary spanning behaviour, boundary spanners can also be facilitated and hindered in their role by other factors (Van Meerkerk & Edelenbos, 2018). The complexity and dynamics of the environmental characteristics are pertinent to boundary spanning behaviour as boundary spanners face environmental uncertainty, diversity, and interdependency. Boundary spanning behaviour can also be impacted by conflicts that can arise due to issues in role definition and role stressors. Boundary spanners can encounter role conflict, role ambiguity, and role overload, and coping with these issues can affect their performance. Furthermore, organizational support and feedback may not only affect spanning behaviour but can also impact their satisfaction, motivation, and commitment. As boundary spanners are defined by their role rather than their organizational function, conflicting demands and needs of different stakeholders may arise. Organizational support in terms of management backing them, empowering them to make certain day-to-day decisions, and giving feedback on their performance as well as the dynamics with co-workers are essential to cope with these demands and needs. Depending on the situational demands and personal capacities, the various tasks of boundary spanners can be combined in a profile of fixer, bridger, broker, or innovative entrepreneur (Van Meerkerk & Edelenbos, 2018). The focus of the current study was on individuals who facilitate cooperation across boundaries with the aim of increasing the size of the user group so that teachers across all institutes will engage with the inter-institutional community. We therefore defined boundary spanners as brokers who ‘can facilitate access to novel information, or resources, facilitate transfer of knowledge, and co-ordinate effort across the network’ (Long et al., 2013, p.2).

**Fig. 1:**
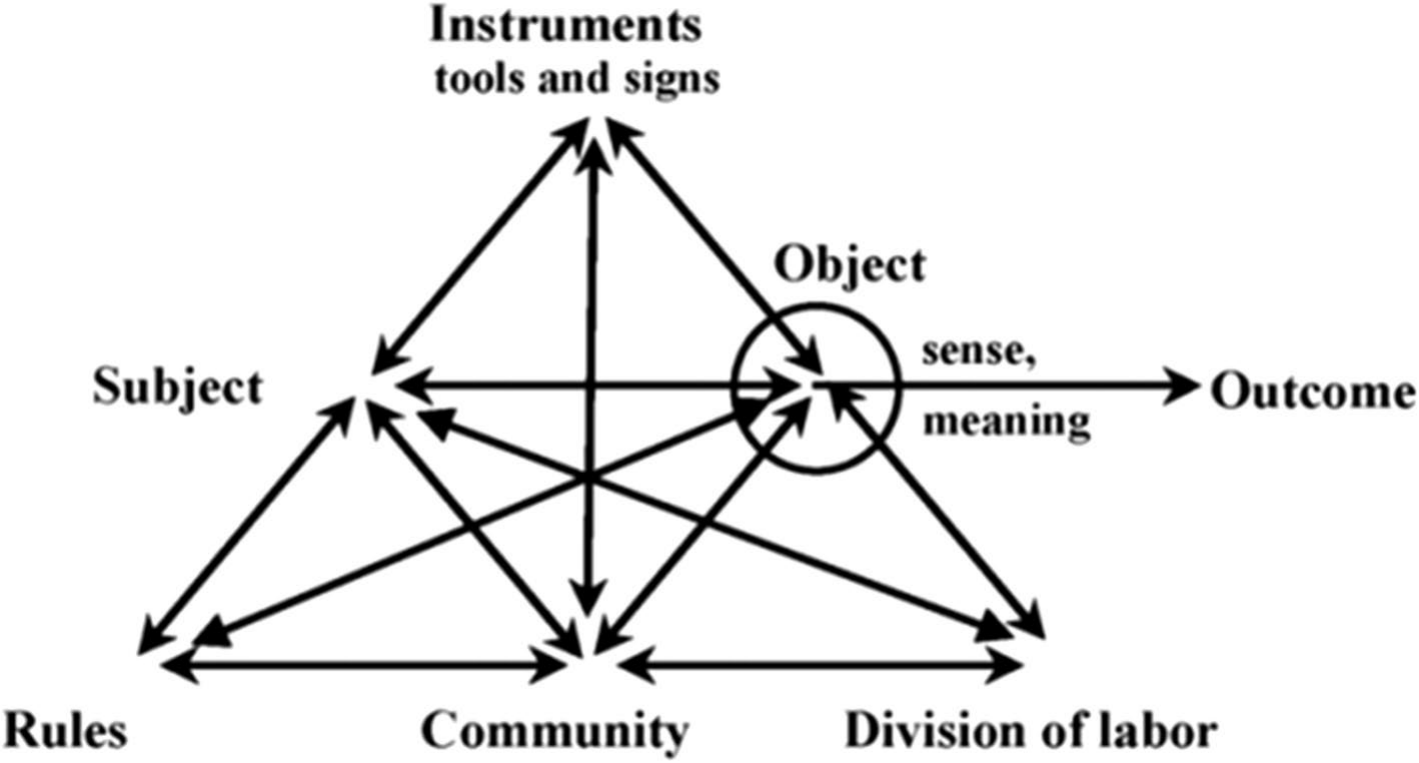
General model of an activity system (Engeström, 1987, p. 78)

Although these studies provide valuable insights into the role of boundary spanners, it is important to note that our understanding of boundary spanning mainly derives from organizational theory. Within the context of higher education, previous studies have mainly explored boundary spanning roles in university-industry collaboration (Corsi et al., 2021; Martin & Ibbotson, 2021; Oonk et al., 2020), within transnational partnerships (Bordogna, 2019) and university-school partnerships (Akkerman & Bruining, 2016; Nguyen, 2020), as well as the role of leaders as boundary spanners (Prysor & Henley, 2018), but little is known about boundary spanners within inter-institutional collaborations. Hill (2020) examined boundary spanning behaviour of brokers intended to connect their campus with the wider network of institutes, but the focus of these brokers was on exploring and transferring the value of the network to their own campus. In the current study, the focus of the brokers was on expanding participation in inter-institutional communities in higher education, a topic on which Hill suggested further research is needed. Thus, to gain a better understanding of brokers’ spanning behaviour, we will explore the actions and perceived impact of brokers’ boundary spanning within the social setting of an inter-institutional community using OER. As the brokers were fulfilling a role within a complex social setting, we used cultural-historical activity theory (CHAT) as a valuable framework, given that goal-directed actions can only be interpreted within the background of the entire activity system (cf., Engeström, 2001). We therefore drew upon the second generation of CHAT as it enabled us to focus on the complex interrelations between the brokers as a subject and the collective activity (Engeström, 2001). Engeström (1987) presented a general model of an activity system (Fig. 1) which provides a framework for exploring the relationships and transformations between different elements of the activity system from the perspective of a subject, which in our case was the broker.

The object is directed at the activity and can be transformed into an outcome through the use of instruments. This process is controlled through sociocultural factors relating to the rules, community, and division of labour in the activity system. The oval in the figure indicates that ‘object-oriented actions are always, explicitly or implicitly, characterized by ambiguity, surprise, interpretation, sense making, and potential for change’ (Engeström, 2001, p.134). The object of any activity is always internally contradictory and these internal contradictions ‘find their outward expressions in external ones’ (Engeström, 2015, p.70).

### Contradictions as a driving force for transformation

Contradictions are defined by Engeström as ‘historically accumulating structural tensions within and between activity systems’ (2001, p.137) and are needed for an activity system to develop. Articulating and overcoming contradictions may catalyse change, whereas unresolved contradictions can obstruct the development of the activity system. Engeström (1987) discerned four levels of contradictions: primary, secondary, tertiary, and quaternary. Primary contradictions appear *within* any of the nodes, for example within rules or within instruments, whereas secondary contradictions occur when there is tension *between* nodes within a single activity system. Tertiary contradictions happen when a newly established mode of the activity system clashes with remnants of the previous mode of activity while quaternary contradictions take place when the main activity system clashes with a neighbouring activity system. Based on Cox’s (2016) research on higher education teachers’ contribution or non-contribution of OER to an institutional repository, we provide some examples of contradictions on each of these four levels in Table 1.

**Table 1:** Examples of contradictions within an OER context as observed by Cox (2016)

Level	Contradiction	Example of Cox (2016)
Primary	Appear within nodes	Within the node Division of Labour teachers, especially those who did not contribute OER, considered teaching in the classroom as their most important role and sharing resources was seen as additional work
Secondary	Appear between nodes	A key contradiction appeared between the nodes Object and Rules as teachers were concerned about the possibility that poor quality resources might negatively reflect upon the institute but no guidelines on the quality of resources were provided
Tertiary	Appear between an old and a more advanced activity system	Teachers experienced a clash between the old system of having resources available to only the students in the classroom to the new system in which resources are open to all
Quaternary	Appear between the main and a neighbouring activity system	Teachers experienced a contradiction with the neighbouring activity system of doing research besides the new main system in which they had to spend extra time on preparing quality OER to share in the repository

Contradictions are not directly observably nor directly accessible in empirical data (Harvey & Nilsson, 2021; Kaatrakoski et al., 2017) but can be found through manifestations (Engeström & Sannino, 2011). Nevertheless, it is important to distinguish between conflict experiences and developmentally significant contradictions as ‘the first are situated at the level of short-time action, the second are situated at the level of activity and inter-activity, and have a much longer life cycle.’ (Engeström & Sannino, 2010, p.7). Within the context of this study, we explicitly focused on the experiences of the brokers on the action level. The focus was therefore on the conflict experiences rather than contradictions, although these conflict experiences might indicate possible contradictions.

### This study

Within the domain of open education, CHAT has been applied to explore students’ perspectives when co-authoring OER (Hodgkinson-Williams & Paskevicius, 2012), to understand teachers’ practices with an institutional OER repository (Cox, 2016; Porter, 2013), to identify tensions that teachers encounter when learning how to use OER (Kaatrakoski et al., 2017), and to examine faculty-librarian OER partnerships (Yao, 2020). Yet no studies have examined the role of brokers in the process of cultivating an inter-institutional OER community while, as our introduction made clear, brokers are essential to spanning boundaries across sites. Hence, the focus of this descriptive qualitative study was to illuminate the role of brokers in the process of cultivating an inter-institutional community in higher education. CHAT offers a conceptual framework to analyse the role of the broker within the entire activity system and allows researchers ‘to analyse the past, present and future of the activity’ (Engeström & Sannino, 2021, p.8). Since we were interested in the role of brokers in transforming the activity, the first research question aimed to depict the role of the brokers within the complex social setting they are operating in. The first research question was:

1. What is the role of brokers within the collective activity system of cultivating an inter-institutional community around OER?
  The inter-institutional community was initiated to create a new practice in which institutes would collaborate sustainably. Brokers undertook several actions within the institutes so that culturally new patterns of activity could be produced. The second research related to this:
2. What actions do brokers undertake to cultivate an inter-institutional community around OER and what impact do these actions have on the activity?
  The actions of the brokers were intended to transform the activity, yet ‘this movement is driven by recurring disturbances and troubles’ (Engeström & Sannino, 2021, p.11). Since our focus was on the action level, our research question aimed to gain more insights into the conflict experiences rather than the contradictions that might exist within the activity systems of the institutes. Thus, our third research question was:
3. Which conflict experiences do brokers encounter in their role of fostering sustainable collaboration on OER among higher education teachers across institutes?

## Method

### Research context

There is a strong focus on OER within higher education at policy level in the Netherlands (OCW, 2019). In this descriptive qualitative study, we explored a project in which 15 universities of applied sciences (UAS) collaborated on sharing knowledge and resources. The project was initiated with funding from a national program on open online education. Two categories of institutes can be distinguished within this ‘Together Nursing’ project. Seven institutes received funding for specific tasks, and they will be referred to as core institutes. The remaining eight institutes did not receive funding and will be referred to as project institutes. Brokers were appointed from all 15 institutes to act as spanners between the project and the institute. Brokers took up this role alongside their regular role as teachers and, in some cases, also as health care professionals.

### Data collection

Before commencing the research, ethical clearance was given by the Research Ethics Committee of ICLON-Graduate School of Teaching at Leiden University. After gaining approval from the project manager to invite the brokers to participate voluntarily, we sent out information letters with details about the study. The first and second authors were responsible for data collection. The first author was an outsider to the research context while the second author was involved with the project. A variety of data sources were used to enhance our understanding of the details of the role of the broker.

#### Documents

Documents that were created before and during the course of the project were accessible to the researchers. They consisted of the project plan, a mid-term evaluation report, quality rubrics, and minutes of meetings. A total of 38 documents were reviewed, of which 33 min of meetings.

#### Process reports

As part of the project, brokers were asked to complete a pre-structured process report after each period (approximately every 2 months). In these reports, brokers were asked to give an update on the progress of the project objectives, any issues within the institute that might impact these objectives and to what extent the broker was satisfied regarding the familiarity with and use of the project within the institute. The project manager used these reports to monitor progress and to gain insights into issues within the institutes.

A total of 89 process reports were completed across nine periods. Table 2 shows the number of reports divided across both core institute and project institutes.

**Table 2:** Number of process reports received by both core and project institutes

Period	Core institutes (*n* = 7)	Project institutes (*n* = 8)	Total
1	3 reports	4 reports	7
2	7 reports	8 reports	15
3	7 reports	7 reports	14
4	6 reports	6 reports	12
5	1 report	2 reports	3
6	7 reports	7 reports	14
7	7 reports	4 reports	11
8	2 reports	6 reports	8
9	1 report	4 reports	5

#### Focus group

The initially planned focus group with the core brokers was cancelled last-minute due to the outbreak of COVID-19 and was replaced by an online focus group. To minimize the workload of brokers during this hectic time, they were advised that if an institute had more than one broker, it would be sufficient if one broker could participate. Brokers from all seven core institutes agreed to voluntarily participate.

The focus group concentrated on the brokers’ experiences and reflections in their role as broker. After an introduction about the goal of the focus group, we posed several questions to start the conversation. For example, ‘Looking back, what went well?’ and ‘Were there aspects that did not go as planned?’ Triggers were used if needed to encourage brokers to elaborate on their answers. To prepare the brokers for the focus group, a reflection report was distributed among the participants beforehand (see ‘Reflection reports’). Table 3 presents the pseudonyms of the core brokers that participated in the online focus group and whether they completed the reflection report.

**Table 3:** Demographics and pseudonyms of the participating core brokers in the focus group

Broker	Gender	Reflection report
Jack	Male	Yes
Sarah	Female	No
Chloe	Female	Partly
Xander	Male	Yes
Tony	Male	No
Kim	Female	Yes
Michelle	Female	Yes

Due to the necessity of holding the meeting online, information regarding data handling and the goal of the meeting was communicated beforehand. The focus group itself lasted approximately 45 min. The verbatim transcript of the focus group was sent for member check. No additions or changes were requested.

#### Reflection reports

Brokers completed a reflection report at the end of the project. In it they reported which actions they had carried out were (a) the most valuable and (b) the least valuable, as well as to what extent they were satisfied with the use of both (c) the OER repository and (d) the online community within their institute. As the project brokers did not meet in a focus group, they reported on two additional questions in which they were asked about (e) their experiences as a broker and (f) what is needed to achieve sustainable collaboration. Again, where an institute had more than one broker, a (collective) response was requested by one broker. A total of five (out of seven) core brokers and five (out of eight) project brokers submitted a report. No pseudonyms were given to the project brokers.

### Data analysis

The collected data were analysed in five steps. The first step focused on condensing the process reports and minutes. No data were excluded from further analysis in this step. For the process reports, close-ended questions were aggregated in tables while all open-ended questions were copied verbatim. This resulted in 15 overview documents, one for each institute, rather than 89 separate process reports. The minutes were organized chronologically in one Excel file based on the composition of the group. Rather than 33 separate documents, we now had one document that could be used for further analysis.

The second step was designed to describe the context in which the brokers were positioned. The project documents were analysed, and codes based on the elements of the general model of an activity system (Engeström, 1987) were assigned to fragments in the documents. After agreement on the description of the activity system by the first two authors, validation by the project manager was requested. This led to some small textual changes.

In the third step, the minutes of the meetings were analysed. This led to an overview of topics that were discussed during the course of the project. Subsequently, we used these topics to code the brokers’ open response answers in the process reports. Within each topic, subcoding was used to code the different actions carried out by the brokers during the course of the project.

In the fourth step, the qualitative data from the focus group and the reflective reports were connected to the elements of CHAT. The selection of these fragments was wide-ranging so that the richness of the data was maintained at this stage. Then, the first cycle of coding was started (Miles et al., 2014). We used evaluation coding to note whether brokers made a positive or a negative remark. Negative remarks indicated perceived resistance or opposition, while positive remarks indicated perceived approval or acceptance. A neutral code was used for remarks that could not be classified as either positive or negative. The evaluation coding was complemented by descriptive coding (to note the topic) and subcoding or in vivo coding (to note qualitative evaluative comments). In this step, therefore, we specifically focused on and selected brokers’ positive and negative remarks regarding actions and perceived impact. It is important to note that the focus was on illuminating the brokers’ experiences within their own activity system; frequency of actions and impact were therefore ancillary.

Finally, in the fifth step, the second cycle of coding applied axial coding to examine the relations and interactions of the elements of the activity system. We deepened our analysis of step four to explain brokers’ conflict experiences during their efforts to transform the activity. As Engeström (2001) argues, the ‘object-oriented actions are always, explicitly or implicitly, characterized by ambiguity, surprise, interpretation, sense making, and potential for change’ (p. 134). This second cycle of coding enabled us to link data across elements and thereby illuminate the brokers’ conflict experiences within the temporary activity system.

The first and second authors led the first and second cycles of coding. Differences in coding were discussed in the research team until consensus was reached.

## Findings

### Past, present, and future of the activity

As it is important to take the history of the object into accounts as it impacts how it is interpreted by the people engaged in the activity, we describe the historical activity system and the desired future activity system in this paragraph. It was hoped that the desired system would have evolved by the end of the temporary project system ‘Together Nursing’ in which the brokers were operating.

#### The historical activity system vs. the desired activity system

In the historical activity system, all institutes operated independently of each other regarding teaching practices and resources. Of course, teachers might have collaborated across institutes in this historical activity system but, if they did, it was either hidden, incidental, or informal. An opportunity to extend collaboration across institutes arose in 2012 when a new professional profile was presented by the professional nursing association. This led to a collaboration across institutes (united under the umbrella of the National Consultation on Nursing Education (LOOV)), which resulted in a collaboratively designed new curriculum called Bachelor of Nursing 2020 (BN2020) in 2016. Around the same time, the Ministry of Education launched a grant program for 1-year projects to explore the creation and sharing of OER across institutes. BN2020 offered an ideal context since (1) it provided institutes with a common language and (2) new topics in the curriculum compelled institutes to develop new resources. Subsequently, in 2017, a pilot project was instigated by five institutes to explore opportunities for collaboration and possible technical infrastructure (OER repository and online community). Due to the success of this project, it was decided to continue and extend the collaboration to all institutes that offer BN2020. Thus, a temporary project system was initiated to realize the desired future activity system in which sustainable collaboration between institutes on sharing practices, knowledge, and OER within the nursing discipline would be accomplished. This project, called Together Nursing, that ran from 2018 to 2020, was the focus of this study.

#### The present activity system

**Fig. 2:**
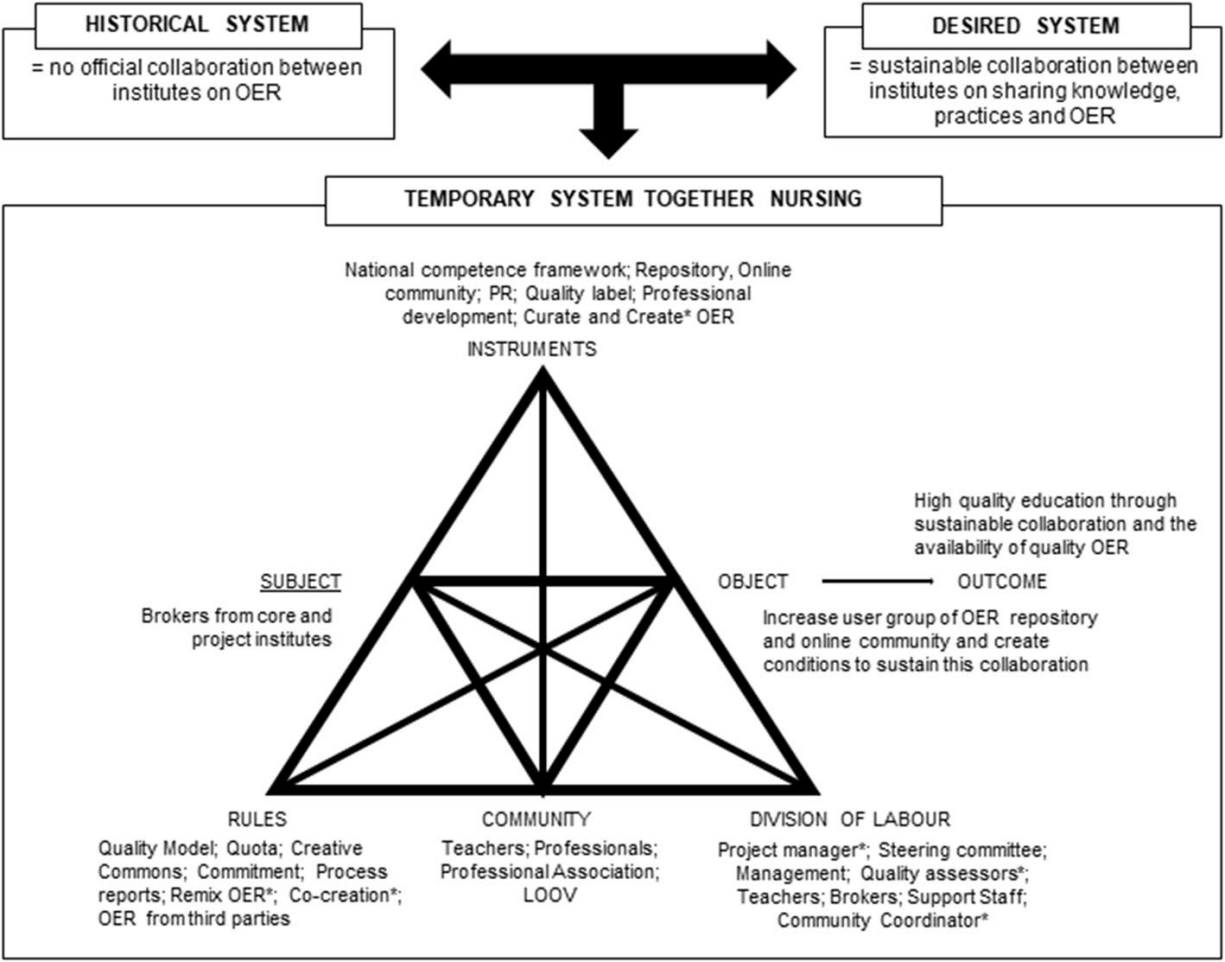
A visual representation of the context in which the brokers were operating. Specific tasks of core institutes specified by an asterisk

We investigated the perspectives of the operating brokers within the present activity system. A visual representation of the elements and interrelationships of this activity system is presented in Fig. 2. This section provides a description of the present activity system, but a more detailed description is available in Online Resource 1.

Brokers were operating in the activity system to endorse the project objectives within their institutes. Their actions were shaped by the object of the temporary activity system which was (a) to expand involvement in the sharing and reuse of high-quality OER and participation in the online community to teachers across all 15 institutes; and (b) to create structures and conditions to foster the sustainability of the collaboration after the project period. Brokers applied mediating instruments within their institute to turn the object into the desired outcome. Brokers for example, applied different means to encourage teachers to engage with the OER repository and the online community, including professional development, advertising and mailings, and curation of OER. However, brokers are part of the collective activity; thus, interaction between subject and object is not only mediated through instruments, but also by the interrelations between the community of actors in the activity system who share the general object; the implicit and explicit regulations, norms, conventions and standards that constrain actions; and the division of labour between actors in the community (Engeström, 2001). The community comprised of approximately 600 participants, mainly teachers from the 15 institutes. Collaboration was sought with the professional nursing association as well. The community shared the outcome of high quality education through sustainable collaboration and the availability of quality OER. Brokers interacted with the community, but at the same time certain rules were imposed in this temporary activity system which impacted the actors in the community. For example, each institute was allocated and committed to share a specific number of OER (quota); a quality model had been developed and adopted which provided teachers with guidelines to optimize the quality of their resources; and brokers attended frequent evaluation moments to discuss progress and possible issues within the institutes. These explicit regulations and standards shaped the actions of everyone in the community, including the brokers as it deviated from the traditional way of working. Brokers also had to navigate both the ‘horizontal division of tasks and the vertical division of power and status’ (Engeström & Sannino, 2010, p. 6). The activity was organized according to the division of labour distributed across all 15 institutes, although the core institutes had more responsibilities than the project institutes. Within the institutes, management had given their commitment to the present activity system and the desired outcome. The project manager had the coordinating role in the project by monitoring progress and disseminating knowledge, and the project itself was overseen by a steering committee which could intervene if progress within an institute stalled. Quality assessors assessed the OER in the repository on the indicators of the quality model and, if the OER complied with them, they awarded a seal of approval. To foster sharing and reuse of OER, teachers were supported by support staff (e.g. library, ICT, or educational support).

Conclusively, the analysis of the present activity system stresses the interrelations between elements of this complex reality in which the brokers were operating. Brokers aimed to transform the collective activity through their actions which we discuss in the next paragraph (‘Brokers’ actions and impact’), albeit this did not occur without conflict experiences which we discuss in paragraph ‘Brokers’ conflict experiences’.

### Brokers’ actions and impact

The object of the brokers was to increase the user group of the inter-institutional community around OER and to create conditions to sustain this collaboration. Brokers’ experiences of their actions and the impact of those actions are presented and illustrated in this section.

#### Brokers’ experiences of their actions

Brokers enacted several instruments to encourage teachers (i) to engage with the inter-institutional community, (ii) to use the OER repository, and (iii) to use the online community. Additional actions were aimed at (iv) creating the necessary organizational structures. An overview of the actions as executed by the brokers is provided in Online Resource 2.

Brokers initially used advertising, mailings, and large-scale meetings to encourage teachers. These instruments enabled them to reach a large number of teachers, but due to difficulties they experienced getting teachers to engage with these instruments, brokers shifted to small-scale, personal, and content-oriented approaches. For example, Kim explained: ‘In the beginning, we mainly organized some larger meetings. First meetings within the educational programs, then in the various teacher teams. The more it became individual, in groups of six but indeed also individual like ‘hey, I’ll bring you up to speed, come and sit down’ […], the more it became widely supported’. Professional development was also used by the brokers to offer teachers support (sometimes one-to-one) to engage with the inter-institutional community.

Other actions were specifically directed at the creation, sharing, and reuse of resources in the repository. For example, to foster reuse, brokers showed relevant resources that aligned with teachers’ teaching content or they stressed the relevance of the repository for curriculum reforms. To foster sharing, brokers scheduled plenary sessions to share OER, applied the metadata form, or uploaded OER for teachers themselves. Actions that aimed to invite teachers to voluntarily share resources on their own (e.g. open call, stress the quota) were experienced as less successful. For example, one broker explained that she herself would ‘actively search for beautiful resources in the digital learning environments to share [in the OER repository]. I would recommend this method to everyone, instead of focusing solely on the quota. It is much more rewarding to look at what colleagues do in their classes and to share the best components with colleagues at other universities of applied sciences’.

Other actions aimed to cultivate the online community. Brokers emphasized the value of the online community among teachers by explaining its relevance to their teaching content and practice. As one project broker stated: ‘Teachers need to get a clear picture of “What’s in it for me? Does it make my job more efficient? Easier? More fun?” Then they’ll be willing to participate’. An action that brokers would like to have included was to also extend the online community with face-to-face meetings. Kim made clear that teachers expressed ‘a need to see who you’re collaborating with’ but this was not possible due to the COVID-19 pandemic.

Additional actions were directed at structuring the division of labour within both the project organization and the organizations of the institutes. For example, brokers stressed the importance of the role of the project manager, the quality assessors, their role as brokers, and other enthusiastic persons within the project organization. Chloe made this clear by saying: ‘I think that the broker role is a crucial factor. You also need a good project manager, but the broker’s role is so essential. Yes, […] you need a driving force who encourages people based upon their own enthusiasm’. Brokers also directed their actions to realize new structures within the institutes. Brokers were positive about the pre-conditions they had created that would contribute to the new activity. Collaborations with the libraries were initiated and teachers’ engagement in the inter-institutional community was integrated into HR interviews. Yet, at the same time, a few brokers stated that it did take much more time than expected to create the necessary pre-conditions within the institute and that the collective responsibility should have been stressed earlier on. Xander explained this by saying: ‘I think that we could have done a better job of explaining within the team how we would attain the number of open resources. That doesn’t take away the fact that everyone was enthusiastic about the project. I think that this […] has been emphasized more than the collective responsibility of sharing resources’.

#### Impact of brokers’ actions

The goal of brokers’ actions was to transform the collective activity. In relation to the object of the temporary activity system, brokers stated that enthusiasm for the Together Nursing project was commonly expressed by teachers and by nursing professionals alike. Brokers felt that their actions to encourage teachers to engage in the inter-institutional community did indeed lead to an increase in teachers using the OER repository and the online community. Teachers used the repository to find resources or to gain inspiration. Kim illustrated this by saying: ‘[I could] give an example of a clinical reasoning lesson that was approached in a specific manner by some colleagues. They used lessons with different approaches [from the repository] to provide students more custom-made lessons’. The online community provided a place to connect and share practices, insights, or questions. Xander explained that this led to a shared problem space: ‘I thought I was the only one in the country who was facing this problem […]. And now all of a sudden, I know that, well, almost all universities have this problem’. Additionally, brokers noted that barriers between institutes diminished, resulting in a strengthened collaboration across institutes. For example, Sarah explained that: ‘without coordination, new collaborative projects would not have come into being […]. Collaboration has been achieved and the […] limitations or the barriers to not only having a look at the other [institutes], but to also using them or to having the confidence to create something together, seem to be falling away. It happens more quickly and easily’.

In addition to the intended transformation, brokers mentioned that their actions also impacted teachers and institutes in other ways. They stressed, for example, that teachers gained an increased awareness of the outline of the curriculum. Sarah explained that: ‘this project has contributed that […] people not only look […] within their own subject area but also look at how it relates to other lessons. I [notice] that people have an increased awareness of the entire curriculum and [they] also notice if there is something missing, if something should be added or if there are possibilities for changes’. Within the institute, brokers explained that the adoption of the quality model resulted in a conversation within the institutes about quality. As Tony explained: ‘Those [quality] criteria have been accepted by our curriculum committee, the curriculum council, and they actually use it to assess new courses […]. What do we consider quality? What do you check? That [conversation] has become a lot more introspective’.

#### Core versus project brokers

We can deduce from the brokers’ individual experiences that it was difficult to encourage a large number of teachers to engage with the inter-institutional community around OER. A small-scale approach was perceived as the most successful. Both core and project brokers experienced the set quota (rules) as a hindrance. Actions that aimed to invite teachers to voluntarily share resources on their own were not that successful, which resulted in brokers taking up this task themselves. However, a difference in attitude regarding these rules became evident. Whereas the core brokers agreed that the top-down quota was an impediment, they also emphasized that it was a means to make the yielded deliverables transparent. Or as Michelle stated: ‘When you receive grant money and therefore hours, […] I consider it very reasonable and normal that you are also obliged to show that you work for […] the project. And the most tangible thing is that you ensure that educational resources are shared. […] And do I like doing it? No, but I do see why and I also think it is justified’.

When comparing the impact of brokers’ actions as perceived by the core brokers versus the project brokers, a sharp contrast was discernible. Whereas core brokers described several positive impacts of their actions, the project brokers were more negative. The only positive impact they mentioned related to the enthusiasm among teachers and their awareness of the existence of the repository and the online community. Moreover, core brokers seemed to be more conscious of the fact that the realization of the desired activity system takes time. Michelle for example stressed her experience *that* ‘I do think it is also something that we’ve all experienced […] that there is a really very long running-in period’. And Kim explained that they made the conscious choice to take one step at a time: ‘We said okay we have now participated with the grant application and the [corresponding] deadline. We’re just going to focus on that deadline right now […] and after that we will focus on the sustainability’.

### Brokers’ conflict experiences

Brokers encountered several conflict experiences while executing the different actions to cultivate the inter-institutional community. This section presents these perceived conflicts in which we refer to the elements of the activity system as presented in Fig. 2.

Although brokers reported an impact of the inter-institutional community around OER on teachers’ practice, they experienced conflicts as they felt that their actions had not led to a major transformation of the teachers’ entire work activity (object). Brokers mentioned that use of the repository was limited and that willingness to share resources was still a major impediment. As one project broker explained: ‘Colleagues do not use [the OER repository] and also prefer not to share. They are still afraid that others will run off with their ideas and [they] don’t want to be convinced of the fact that there are always rights attached [to their resources]. Colleagues do not take the time to search and look around [the OER repository]’. The same applied to use of the online community. While the online community did foster knowledge sharing and exchanges of practices, brokers reported that not all teachers made use of the online community. In particular, a number of specific theme groups were frequently used by teachers from different institutes, but as one broker stressed: ‘Few teachers participate in the [online] community and they indicate that they have no need for it. Where there is a need […] people will connect with each other. […] but teachers who do not have a specific area of interest or responsibility within the education program do not see what the community can offer them. No matter how much you promote it’.

Brokers not only reported that the new activity was not widely endorsed within the institutes, other conflict experiences relating to elements of the project activity system also emerged. Brokers struggled, for example, with the ambiguity and the responsibilities of their role (subject). Michelle explained this by stating that ‘Well I think as far as I’m concerned that distinction between the broker role and the project leader role was indeed quite ambiguous within our institute’. Brokers also felt the pressure of their responsibility. As Chloe explained: ‘If other people don’t take up their task, I will. That’s my downfall, but this project has shown over and over again that this is very difficult. If you delegate something to other people, will it happen?’ This tension in the broker role was amplified due to the quota imposed by the project (rules). For example, Kim explained: ‘First create the support capacity and FTE at the support staff (such as the library) before making concessions on the quota. The project must be broadly supported. I was largely responsible and on my own’. Jack also illustrated the consequence of this quota by saying: ‘What’s been difficult from the beginning, is that the project within our institute had a bit of a top-down approach. It seemed like, in our case [colleague] came up with numbers and targets every quartile that we had to meet. Which made it seems like we were a project in the name of the management’. At the same time though, coordinating with management to plan actions to realize the intended transformation was an issue (division of labour). Tony illustrated this dilemma by sharing his experience: ‘What I ran into very much was that […] it shouldn’t just be between quick contacts. Do you have something for me? There also has to be a commitment from the team […]. And the annoying thing was that the management gave their commitment, […] but the moment you say”guys what are we going to do now?”, it was all toned down like”no [teachers] shouldn’t feel obliged and they don’t want to”. Well, then nothing happens’. At the same time, brokers were also impacted by organizational changes relating to reorganizations as well as high enrolment of new students which in turn resulted in personnel changes (community and division of labour). These changes were magnified by the impact of COVID-19 on teachers’ practices. Jack explained: ‘We have just gone through a reorganization. We also just had a very high enrolment and the expectation is that the number of students will increase next year as well. And because of that, the number of teachers will also increase. […] If you see right now how [teachers] are overwhelmed in the Covid time with other ways of working, then I really feel sorry for them’.

## Discussion

This descriptive qualitative study set out to illustrate the role of brokers in cultivating inter-institutional collaboration across 15 higher education institutes. We applied CHAT as it offers a conceptual framework for analysing the role of the broker within the background of the entire activity system. Our findings show that brokers used several instruments to encourage teachers to engage with the inter-institutional community, to use the OER repository, and to use the online community. Additional actions were aimed at creating the necessary organizational structures. Brokers concluded that although a wide range of instruments were needed to foster the transformation, the small-scale, personal, and content-oriented approaches to encourage teachers to engage with the OER repository and the online community were perceived as the most valuable. The brokers were key in this regard, since they had the central position within the institute as peer colleagues while also having the expertise to relate to the teaching content. Yet, at the same time the findings show that brokers encountered conflict experiences due to the demanding context in which they were operating, the organizational constraints they were confronted with, the ambiguity and responsibilities of their role, and the limited perceived impact on teachers’ practices. In this section, we will discuss both the theoretical and practical implications for collaboration across higher education institutes that follow from our findings.

### Brokers as boundary spanners

CHAT proved to be a valuable framework for gaining insight into the role of brokers because it emphasizes the sociocultural elements and its interrelations that shape collective actions directed at the shared object. Therefore, CHAT offered ample opportunities to gain a deeper understanding of the elements, and the relations between the elements of the activity system. Figure 2 visualizes the nature and relationships within and between elements. The analyses illuminated that brokers’ actions yielded the intended transformation of the collective activity, albeit to a more limited extent than expected. Brokers were able to apply actions to engage teachers with the inter-institutional community while also acting to create organizational structures, but a major transformation did not occur. The role of the broker was hindered due to conflicts they experienced. Despite their efforts and the enthusiasm that they received from teachers and health professionals alike, brokers noticed that the desired object was still not widely endorsed within the institute at the end of the project. It could be that the expectations were too ambitious to encourage all teachers within the institutes. We therefore align with the recommendation of Akkerman & Bruining, (2016) that specific developmental aims distributed across time should be formulated through which choices can be made about who to involve and when to involve them. It could be more gratifying to focus on willing teachers at the beginning with the hope that good practices would trickle down to other teachers over time. At the same time, a mismatch was often found between practice and institutional responsibility and structures that hindered the transition from conventional teaching to new practices embedding OER (Kaatrakoski et al., 2017). Kaatrakoski et al. therefore stress that organizational change management is critical to encourage teachers to transfer from the historical to the desired practice in which OER and collaboration are part of teaching practice. Even though brokers were able to make changes within the organization by altering the historical-cultural system to the new processes and operations (e.g. by setting up partnerships with the library, by integrating OER into HR interviews), the rules of the project activity system and the limited support from management proved to be impediments to success. Management did not empower the brokers within their role even though it was important that they receive organizational recognition and support to assist them in their role (Akkerman & Bruining, 2016). The brokers’ lack of power was exacerbated by detrimental effects of organizational and societal issues. Reforms within the departments, a high number of new teachers, and COVID-19 influenced brokers’ actions and diverted the focus from the inter-institutional collaboration on OER. Those issues greatly influenced the brokers while they had limited capacity to counteract them. Although not all challenges are easy to overcome, brokers must feel supported in their boundary spanning role. We therefore agree with Prysor & Henley, (2018) that leaders ought to change their leadership to not only focus on leadership within teams but to also include leadership that supports boundary spanning.

In conclusion, brokers were essential in cultivating the inter-institutional community due to the unique positions they held among colleagues even though challenges that must be overcome also emanated from this position. The findings of this study not only provide new insights into the role of brokers in fostering educational change through OER in higher education collaboration, it also corroborates the work of other studies on antecedents of boundary spanning behaviour (Van Meerkerk & Edelenbos, 2018).

### Implications for practice

The main question that arises from our discussion is how brokers can be supported in their role to cultivate collaboration across institutes. The strengths of using CHAT were that it gave us a theoretical lens with which to examine the complex and evolving activity system in more detail. It enabled us to examine the brokers’ actions, but it would be of interest to also explore other perspectives (subject). The conflicts that brokers encountered derived partly from the clashes of views that sometimes occurred between brokers, managers, support staff, and teachers. It is essential, therefore, to address the multi-voicedness of the object by discussing it regularly with all stakeholders since ‘expansive learning is an inherently multi-voiced process of debate, negotiation and orchestration’ (Engeström & Sannino, 2010, p. 5). If necessary, let go of the initial object and alter it to align it with the local context so that sustainable practices may be realized (März et al., 2017). Additionally, brokers must be aware that although it might appear that actual change in teachers’ practice has been limited, sustainable change takes a long time and actual participation in online communities is always differentiated between a minority of participators and a majority of onlookers (Lantz-Andersson et al., 2018). Even so, only a few teachers prefer online networking (Van den Beemt et al., 2018) and online collaboration in combination with face-to-face meetings would be advised. Finally, brokers encountered role stressors due to the ambiguity and responsibilities of their role. They deployed a plethora of actions to foster change while also setting up needed organizational structures. A broker should therefore be facilitated by the project manager giving clear expectations on tasks, responsibilities, and intended outcomes while simultaneously being provided with time, empowerment, and organizational support from the institute. At the same time, brokers’ role stressors could be lessened if teachers recognized and valued the act of boundary crossing across institutes. We therefore suggest that institutes advocate for collaboration across institutes to follow up on the recommendation of Oonk et al. (2020) that boundary crossing competence be incorporated into teacher competence profiles.

### Limitations and future research

It is important to note that this study had some limitations. First, although some institutes had more than one broker, we decided that it would be sufficient if one broker participated in the study to limit time investment during the COVID-19 pandemic. Even then we were not able to recruit brokers from all institutes since some did not respond to the researcher’s invitation to participate. Because of this we were not able to capture all brokers’ experiences. However, we believe that this limitation was partly ameliorated by combining different data sources and by having a mix of both core and project brokers. Second, this was a reflective study but it would be helpful to examine how brokers’ experiences changed during the course of inter-institutional collaboration on OER. Future research could apply longitudinal designs by, for example, using cyclical interviews, videotaping project meetings, or by using logs to follow brokers up close. It would also be interesting to gain more insight into collaboration between brokers. Third, although this study improved our understanding of the role of brokers within a specific highly contextualized case, we relied on the brokers’ perceptions. It would be worthwhile to further explore the roots of the conflict experiences by shifting the focus from the brokers’ action level to the activity level so that changes within the institutes and in teachers’ practices could be investigated. In that way, contradictions within and between activity system could be substantiated (Engeström & Sannino, 2010).

## Supplementary Information

Below is the link to the electronic supplementary material.

Supplementary file1 (PDF 102 KB)

Supplementary file2 (PDF 120 KB)

## Data Availability

Not applicable.
