# Supplementary material for: The role of brokers in cultivating an inter-institutional community around open educational resources in higher education
Source: High Educ (Dordr). 2022 Jun 3;85(5):999–1019. doi: 10.1007/s10734-022-00876-y (PMC9165544; doi:10.1007/s10734-022-00876-y)
Supplement: Supplementary file 1 — Supplementary file1 (PDF 102 KB) [file 10734_2022_876_MOESM1_ESM.pdf]

Online Resource 1 | Context in which the brokers were operating

To foster the transition from the historical system to the desired system, a temporary activity system was set up to achieve sharing and reuse of OER within an active professional community of teachers across institutes. In this supplementary file, we provide a detailed account of this temporary activity system in which the brokers (subject) were operating.

Object and Outcome

The object *'refers to the "raw material" or "problem space" at which the activity is directed*' (Engeström & Sannino, 2010, p.6). The object of the temporary activity system was: a) to expand involvement in the sharing and reuse of high-quality OER and participation in the online community to teachers across all 15 institutes; and b) to create structures and conditions to foster the sustainability of the collaboration after the project period. This should ultimately lead to the outcome of high-quality education.

Instruments

The brokers used mediating instruments to achieve the object. An OER repository was made available for which a subject vocabulary was developed so that searching, finding and uploading would take place under standardized and recognizable terms. If OER met the requirements as outlined in the quality model, a quality label was given as a seal of approval. This made it easier for teachers to quickly find the right materials of guaranteed quality. In addition to the repository, an online professional community was available for teachers. The aim of this community was to provide teachers with the opportunity to connect, discuss OER and practices or identify the need for new OER. New OER were created by the core institutes based on teachers' needs. To raise awareness of both the repository and the online community, PR resources were available. Additionally, professional development activities took place, since creating, sharing and using OER entails expanding the traditional role of teachers.

Rules

The rules refer to the explicit and implicit regulations and standards that constrain actions (Engeström & Sannino, 2010). Several rules were imposed in this project and all institutes committed to follow them when agreeing to participate in the project:

- A quality model had been developed and adopted. This model provided teachers with guidelines to optimize the quality of their own resources while it also provided them with the confidence that the OER in the repository were of high quality.
- A total of 1900 OER would be shared in the repository, all described in accordance with the quality model. Resources did not necessarily have to originate from the institutes, OER from third parties were shared as well (referatory).
- All resources were to be shared under a Creative Commons license.
- A total of 40 new OER would be developed by the core institutes. Objectives were for two or more institutes to co-create new OER by remixing with existing OER if possible.
- The aim was to realize an active community of practice in which approximately 500 teachers would take part.
- Frequent evaluation moments took place through process reports to discuss progress and possible issues within the institutes.

Community

The community is defined as consisting of all involved who share the same object. The potential community of this activity system consisted of all (approximately 2500) teachers within the 15 institutes. The institutes are united under the umbrella of the National Consultation on Nursing Education (LOOV). Collaboration was sought with the professional nursing association. Towards the end of the project, healthcare professionals were approved to participate to create interaction between institutes and the healthcare profession.

Division of labour

Division of labour relates to the '*horizontal division of tasks and vertical division of power and status'* (Engeström & Sannino, 2010, p. 6). The activity was organized according to the division of labour

distributed across all 15 institutes, although the core institutes had more responsibilities than the project institutes. Within the institutes, management had given their commitment to the project. The brokers acted as the link between the project system and the institute. The project manager had the coordinating role in the project by monitoring progress and disseminating knowledge. The project was overseen by a steering committee which could intervene if progress within an institute stalled. Quality assessors assessed the OER in the repository on the indicators of the quality model and, if the OER complied with them, awarded a seal of approval. A community coordinator was assigned with tasks related to community management. Teachers were supported by support staff (e.g. library, ICT or educational support).
