# Supplementary material for: The role of brokers in cultivating an inter-institutional community around open educational resources in higher education
Source: High Educ (Dordr). 2022 Jun 3;85(5):999–1019. doi: 10.1007/s10734-022-00876-y (PMC9165544; doi:10.1007/s10734-022-00876-y)
Supplement: Supplementary file 2 — Supplementary file2 (PDF 120 KB) [file 10734_2022_876_MOESM2_ESM.pdf]

Online Resource 2 | Brokers' experiences of actions they undertook

Focus of actions	Topic	Description	Remark	Actions	Exemplary quotation
Encourage teachers	Encouragement and awareness	Actions aimed at creating awareness about the project Together Nursing and encouraging teachers to engage with it	+	▪ Small-scale meetings<br>▪ Individual approach<br>▪ Relate to teaching content<br>▪ Relate to curriculum reforms<br>▪ Relate to 'what's in it for me'<br>▪ Rewarding and complimenting<br>▪ Respond to covid-19	*'In the beginning we mainly organized some larger meetings. First meetings within the educational programs, then in the various teacher teams. The more it became individual, in groups of six but indeed also individual like "hey, I'll bring you up to speed, come and sit down" […], the more it became widely supported.'*
			-	▪ Large-scale meetings<br>▪ Use of PR posters<br>▪ Mailing	*'Sending out mails and reminder mails. The mails are quickly archived and not looked at again. The topic does not have priority in busy times.'*
	Teacher support	Actions aimed at supporting teachers in using the online community and the OER repository	+	▪ Workshops<br>▪ Lessons<br>▪ Individual support	*'Different team members need different approaches. It also depends on the stage of the process you're in. In the beginning more general workshops and trainings. Later on, the individual approach was more valuable.'*
			-	▪ Workshops	*'Workshop with the library on open sharing. Output: colleagues were aware, but [there is] little time and effort to adapt [resources] themselves.'*
Use of OER repository	Creation of OER	Actions aimed at the collaborative creation of new OER	+	*No reflective remarks on actions*	
			-	▪ Too late involvement of OER designers	*'I think I would have liked to involve the creators of OER earlier on. By doing so, the group becomes a bit larger which provides a bit more power within your institute to enthusiasm more people.'*
	Share and reuse OER	Actions aimed at sharing and reusing OER	+	▪ Relate to teaching content<br>▪ Relate to curriculum reforms<br>▪ Use of metadata form<br>▪ Upload OER for teachers<br>▪ Schedule plenary sessions to share resources as OER	*'Actively searching for beautiful resources in the digital learning environments to share [in the repository]. I would recommend this method to everyone, instead of focusing solely on the quota. It is much more rewarding to look at what colleagues do in their classes and to share the best components with colleagues at other universities of applied sciences.'*
			-	▪ Open call to share OER<br>▪ Enforce teachers to share an x number of OER<br>▪ Schedule plenary sessions to share resources as OER<br>▪ Stress the quota of OER to be shared by the institute	*'Require teacher teams to share an x number of resources. Positive result: resources to share, colleagues getting excited about the OER repository and the online community. Negative result: resistance to the entire project.'*
	OER Quality	Actions aimed at the adoption of the quality model and corresponding quality label	+	*No reflective remarks on actions*	
			-	*No reflective remarks on actions*	

Use of online community	Cultivation of community	Actions aimed at cultivating the online community within the institute	+	▪ Relate to 'what's in it for me'<br>▪ Relate to teaching content	*'I think [that] the subject groups that emerge within the online community are perceived as valuable. This can act as a force of attraction. Teachers need to get a clear picture of "What's in it for me? Does it make my job more efficient? Easier? More fun?" Then they'll be willing to participate.'*
			-	▪ Lack of face-to-face contact	*'That's what a number of colleagues have passed on, that [they] like to know who they are talking to. That [they] like to have met people as [they] are more inclined to look them up and connect with them in the online community. There is a need to see with who you are collaborating.'*
Organizational structures	Within the institutes	Actions aimed at creating the needed organizational structures within the institutes	+	▪ Collaboration with library<br>▪ Integration into HR interviews	*'A number of preconditions are certainly important, but preconditions are not only a metadata form but also making use of the library for example.'*
			-	▪ Limited focus on collective responsibility<br>▪ Organizational pre-conditions not in place on time	*'I think that we could have done a better job of explaining within the team how we would attain the number of open resources. That doesn't take away the fact that everyone was enthusiastic about the project. I think that this […] has been emphasized more than the collective responsibility of sharing resources.'*
	Of the project	Actions aimed at creating the intended structure of the project organization	+	▪ The role of brokers and project manager<br>▪ Quality assessors of OER<br>▪ Enthusiastic individuals<br>▪ Personal enthusiasm	*'I think that the broker role, I think that it is a crucial factor. You also need a good project manager, but the broker's role is so essential. Yes, […] you need a driving force whom encourages people based upon their own enthusiasm.'*
			-	▪ Tedious project meetings<br>▪ Solely focus on institutional commitment<br>▪ Joining project after 1 year	*'What I take away with me for the future is that commitment alone is not enough. The responsibility must be felt and must be implemented in all levels [of the institute] so that when you make agreements, 134 OER, the teachers don't see it as a burden but know that we have to comply with it. And that we have the opportunity to do so, because we have been given the time for it.'*
	External promotion	Actions aimed at promoting the project Together Nursing outside the participating institutes	+	*No reflective remarks on actions*	
			-	*No reflective remarks on actions*	

Note. + = positive remarks and - = negative remarks in relation to actions
